# A patient develops transient unique cerebral and cerebellar lesions after unruptured aneurysm coiling

**DOI:** 10.1186/s12883-015-0303-7

**Published:** 2015-03-31

**Authors:** Kentaro Deguchi, Yuko Kawahara, Shoko Deguchi, Nobutoshi Morimoto, Tomoko Kurata, Yoshio Ikeda, Tomotsugu Ichikawa, Koji Tokunaga, Nobuyuki Kawai, Kenji Sugiu, Koji Abe

**Affiliations:** Departments of Neurology, Graduate School of Medicine and Dentistry, Okayama University, 2-5-1 Shikata-cho, Okayama, 700-8558 Japan; Departments of Neurosurgery, Graduate School of Medicine and Dentistry, Okayama University, 2-5-1 Shikata-cho, Okayama, 700-8558 Japan; Department of Neurological Surgery, Faculty of Medicine, Kagawa University, 1750-1 Ikenobe, Miki-cho, 761-0793 Japan

**Keywords:** Aneurysm coiling, Contrast media, MRI, MRS, Methionine PET-CT

## Abstract

**Background:**

We describe a case of a very unusual complication following a coiling procedure in which the patient developed transient unique cerebral and cerebellar lesions. Lesions were examined not only by magnetic resonance imaging (MRI) but also by positron emission tomography-computed tomography (PET-CT) and proton magnetic resonance spectroscopy (^1^H-MRS).

**Case presentation:**

A 33-year-old woman presented an incidental 3.7 × 3.3-mm unruptured cerebral aneurysm (CAn) in her basilar artery, which was successfully coiled with balloon assistance. A follow-up brain MRI at 1 and 2 months showed a gradual increase in several white matter hyperintense lesions in the left cerebellar, bilateral occipitotemporal and left parietoccipital lobe during fluid-attenuated inversion recovery (FLAIR). These were the only lesions associated with perfused CAn. However, the patient did not show any additional symptoms such as visual disturbance throughout the entire course. ^11^C-methionine-PET (MET-PET) showed an obvious increase in methionine uptake in the lesion corresponding to enhanced areas with gadolinium-enhanced MRI. MRS showed a decrease in the N-acetylaspartate/creatine (NAA/cr) ratio and a slight elevation of the choline/creatine (cho/cr) ratio and a lactate peak in the lesion. A follow-up MRI at 6 and 12 months showed a gradual decrease in the initial hyperintense lesions in FLAIR without any treatment.

**Conclusion:**

We present a case of an unusual complication after a coiling procedure. Although it is difficult to identify this etiology without a pathological examination, it is importance to increase awareness of such a potential complication arising from coiling procedures, because interventional procedures have become the first choice of treatment for cerebrovascular diseases in many countries.

## Background

Neurointerventional procedures such as aneurysm coil embolization have become a frequent treatment option for a variety of cerebrovascular diseases in many countries [1]. Although less invasive than conventional traditional surgical approaches, neurointerventional procedures are not exempt from complications. We describe a case of a very unusual complication following a coiling procedure in which the patient developed transient unique cerebral and cerebellar lesions. These lesions were examined by three techniques: magnetic resonance imaging (MRI), positron emission tomography-computed tomography (PET-CT), and proton magnetic resonance spectroscopy (^1^H-MRS).

## Case presentation

A 33-year-old Japanese woman first visited Okayama University Hospital in February, 2011 to evaluate numbness in her bilateral lower limbs, a condition that had developed since the age of 29, when she had a cervical spinal cord injury due to a car accident. The patient had no other previous medical history.

General medical and neurological examinations showed no other particular findings except for decreased vibration sensation and increased tendon reflexes in her bilateral lower limbs, which were present 4 years after the car accident.

**Figure 1:**
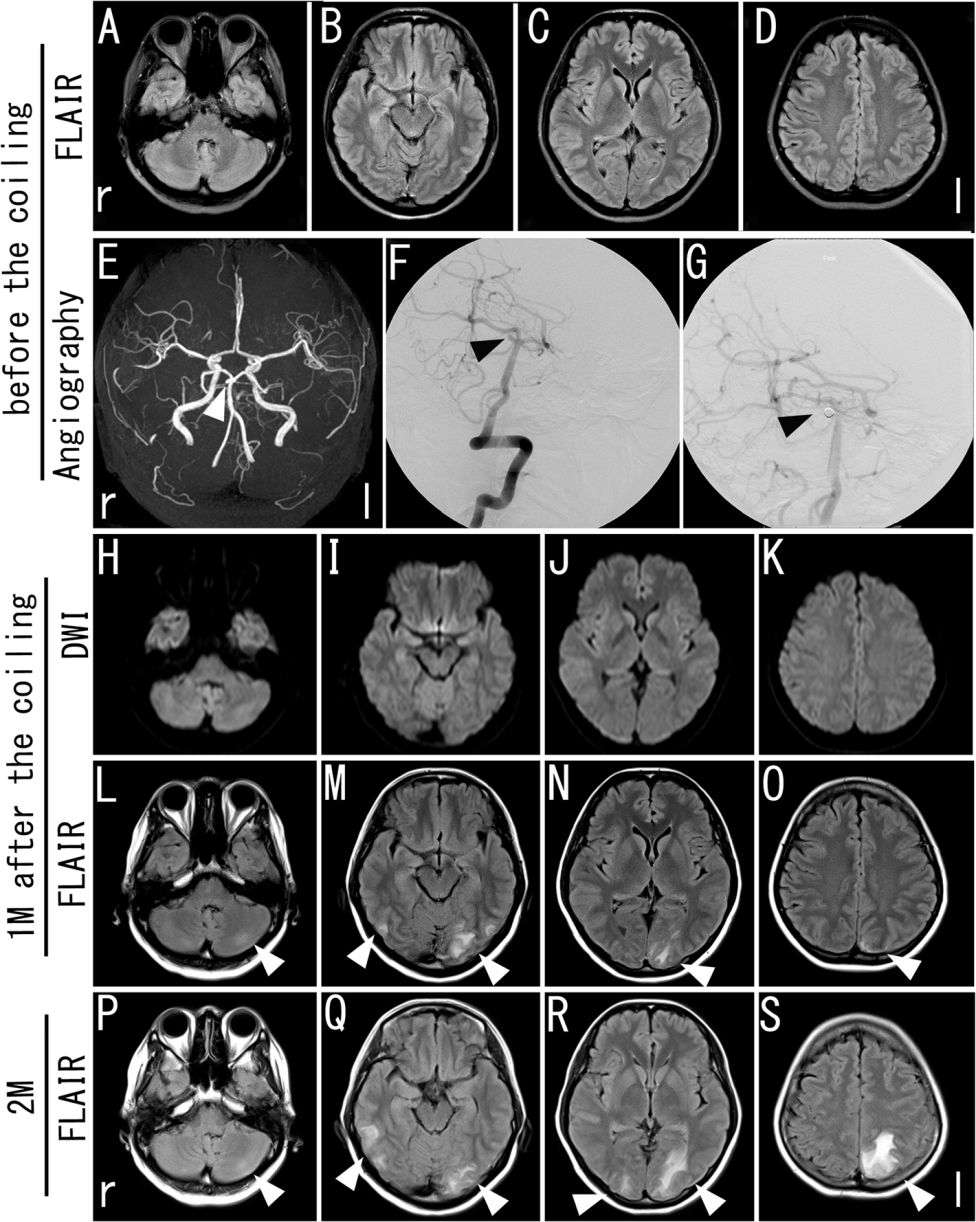
**Findings of brain and spinal MRI, and angiography after the first admission for aneurysm coiling and after aneurysm coiling.** No lesions were observed with axial slices of a fluid-attenuated inversion recovery (FLAIR) image **(A-D)**. Brain MRA showed a 4 × 4 mm unruptured cerebral aneurysm (CAn) in the basilar artery located at the origin of the right superior cerebellar artery (**E**, arrowhead). Precoiling posterior angiography showed an unruptured CAn **(F)** and postcoiling angiography showed the occlusion of CAn and the patency of all vessels **(G)**. After aneurysm coiling, left cerebellar, bilateral occipitotemporal, and left parietoccipital lobe lesions were observed with the axial slices of the FLAIR image (**L**-**O**, arrowheads) without a positive signal of the diffusion-weighted image (**H**-**K**, arrowheads) at 1 month after coiling, which expanded markedly in size at 2 months after coiling (**P**-**S**, arrowheads).

Biochemical blood tests showed no abnormal findings. A brain MRI revealed no lesion in fluid-attenuated inversion recovery (FLAIR; Figure 1A-D). However, a brain magnetic resonance angiography (MRA) incidentally found a 3.7 × 3.3 mm unruptured cerebral aneurysm (CAn) in her basilar artery located at the origin of the right superior cerebellar artery (Figure 1E, arrowhead). When approximately 80 mL of nondiluted iopamidol (Iopamiron, Bayer Healthcare Inc., Leverkusen, Germany) was intravenously injected as the contrast medium (CM), a computed tomographic angiography (CTA) confirmed the CAn. An MRI revealed a small old lesion in the C5/6 level of the spinal cord, which was responsible for her leg symptoms. With extensive informed consent, endovascular coil embolization was performed through the right femoral artery under general anesthesia in which the patient became fully heparinized by 4,000 units of heparin sodium. Approximately 70 mL of nondiluted iopamidol was used throughout the procedure, distributed in one rotational acquisition of 14 mL as 10 standard vertebral injections of 5 mL each (to monitor parent vessel patency and aneurysm occlusion). The aneurysm was framed with a Microplex-10 complex 3/7 coil (Terumo, Isehara, Japan) and filled with 2 Micrus Deltaplush (Micrus Endovascular, San Jose, CA) coils. A HyperForm 4 × 7 balloon (Micro Therapeutics Inc, Irvine, CA) was intermittently inflated and deflated between coil placements. Postcoiling contrast angiography showed complete obliteration of the aneurysm (Figure 1F, G, arrowheads). During the procedure, CM was injected only into the right vertebral artery. The right groin was closed by manual compression. There were almost no changes in vital signs such as blood pressure, heart rate, and respiratory rate during and after the operation. The patient awoke from anesthesia neurologically intact and was discharged from the hospital 72 hours after coiling.

Although the patient did not develop any clinical symptoms, a follow-up brain MRI at 1 month showed several white matter lesions in the left cerebellar, bilateral occipitotemporal and left parietoccipital lobe without a positive signal in the diffusion-weighted image (DWI; Figure 1H-O, arrowheads). A further follow-up MRI at 2 months after coiling revealed the enlargement of each lesion (Figure 1P-S, arrowheads), although she did not show any symptoms such as visual disturbance. Thus, she was re-admitted to our hospital for a detailed examination in July, 2011. However, blood and cerebrospinal fluid (CSF) biochemical tests showed no abnormal findings.

**Figure 2:**
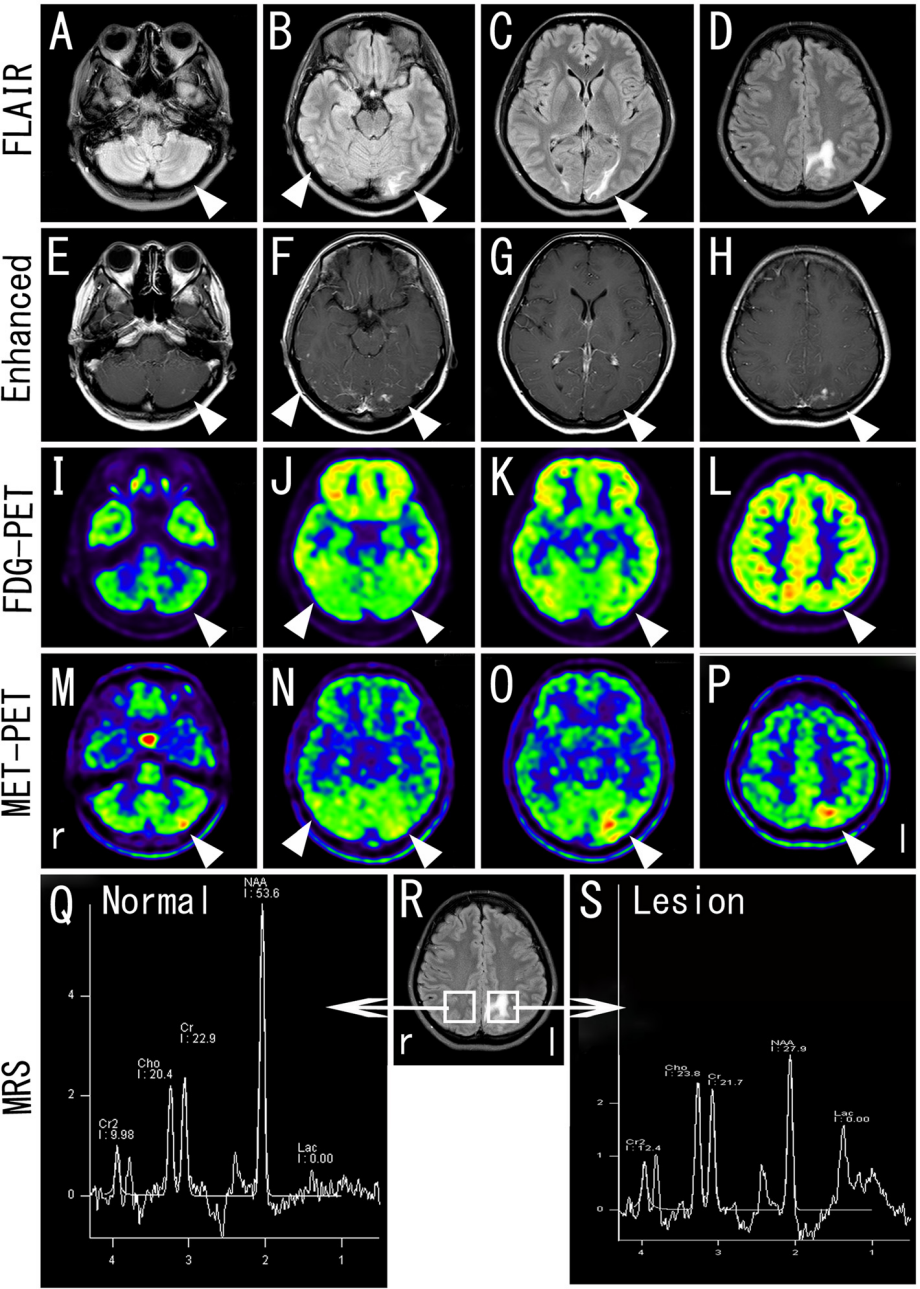
**Brain MRI, PET-CT, and MRS findings at 4 months after coiling.** 2-deoxy-2-[F-18]fluoro-D-glucose (FDG) and ^11^C-methionine (MET) PET studies showed that glucose uptake in the lesion with hyperintense areas by FLAIR image (**A-D**, arrowheads) and enhanced areas by gadolinium-enhanced MRI (**E**-**H**, arrowheads) increased slightly or remained at the baseline level (**I**-**L**, arrowheads), whereas methionine uptake increased (**M**-**P**, arrowheads). ^1^H-MRS showed a slight increase of the choline (cho) peak and a marked decrease of the N-acetylaspartate (NAA) peak with an elevated lactate peak in the lesion **(Q-S)**.

At 4 months, 2-deoxy-2-[F-18]fluoro-D-glucose (FDG) and ^11^C-methionine (MET) PET studies showed that glucose uptake in the lesion with areas enhanced by gadolinium-enhanced MRI (Gd-MRI; Figure 2E-H, arrowheads) increased slightly or remained at the baseline level (Figure 2I-L, arrowheads), while methionine uptake increased (Figure 2M-P, arrowheads). ^1^H-MRS showed a slight increase in the choline (cho) peak with a mean cho/creatine (cr) ratio of 1.10 versus 0.89 in the control. The mean N-acetylaspartate (NAA) peak was reduced with a mean NAA/cr ratio of 1.29 versus 2.34 in the control. An elevated lactate peak was also noted (Figure 2Q-S).

**Figure 3:**
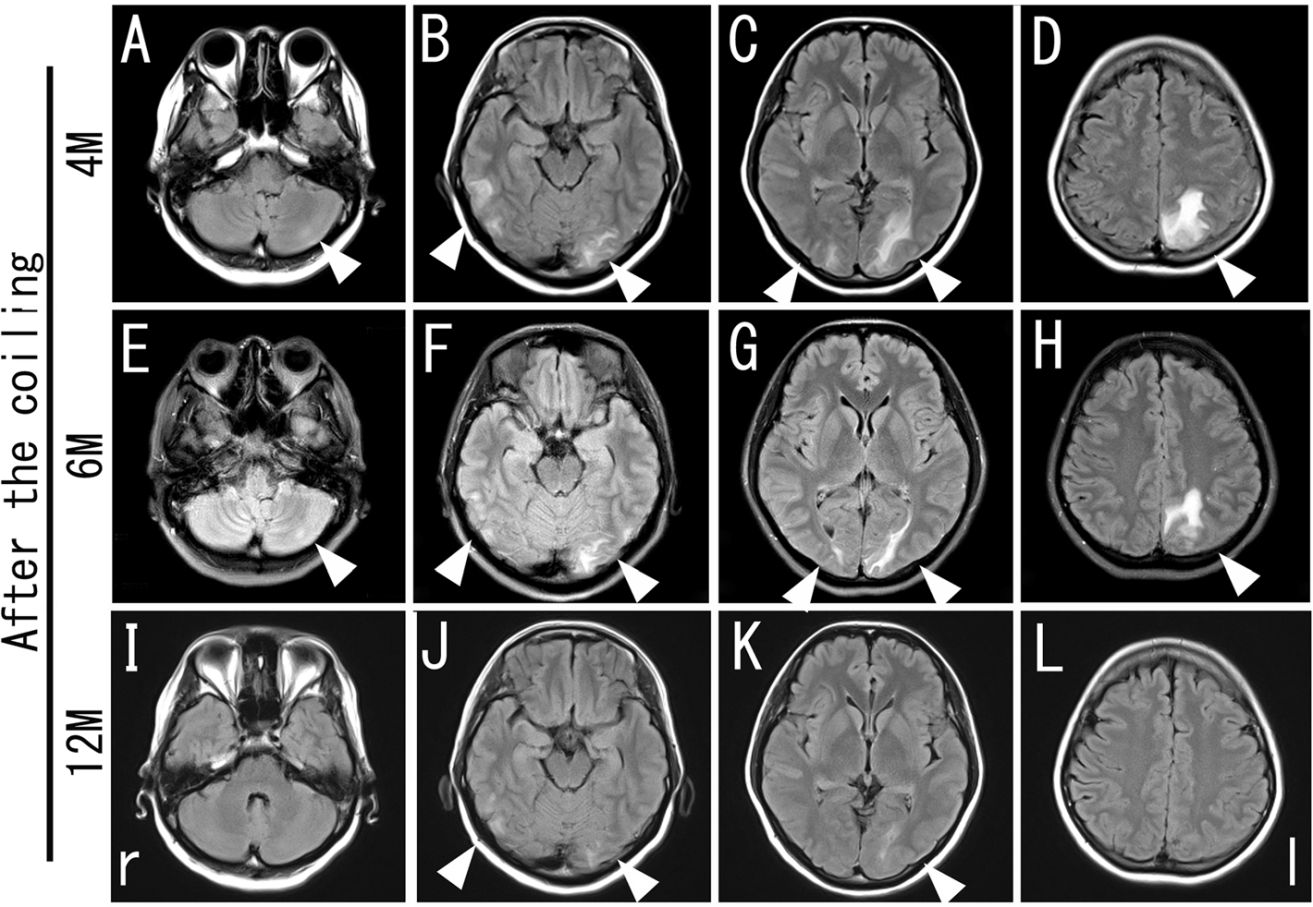
**Chronological changes of the brain MRI after the second admission.** All lesions observed at 4 months after coiling (**A**-**D**, arrowheads) diminished gradually in size at 6 months (**E**-**H**, arrowheads) and at 12 months (**I**-**L**, arrowheads) after coiling.

A follow-up MRI at 6 and 12 months showed a gradual decrease in the initial hyperintense lesions in the left cerebellar, bilateral occipitotemporal and left parietoccipital lobe (Figure 3E-L, arrowheads).

## Conclusions

We report on a patient who presented several asymptomatic cerebellar and cerebral white matter lesions after coiling of the CAn. These lesions recovered completely without any treatments. The occurrence of brain lesions has been described after coiling procedures [2-6]. Hydrocephalus, aseptic meningitis, and perianeurysmal edema are known complications of both bioactive and bare platinum coils used in the treatment of intracranial aneurysms [7]. These inflammatory reactions are believed to be triggered by cytokines involved in the aneurysm healing process induced by coils [8], being proportional to the size of the coiled aneurysm [7]. However, in our case, the pattern of lesions was not perianeurysmal.

Although there was no diagnostic finding in FDG-PET in this case, MET-PET showed an obvious increase in methionine uptake in the lesion corresponding to the enhanced areas with gadolinium-enhanced MRI (Figure 3M-P, arrowheads). Although methionine does not accumulate in normal brain tissue [9], it can at certain stages of stroke, demyelinating disease, and other instances [10-15] due to the uptake of methionine into the macrophage/microglia and reactive astrocytes, and passive spreading as a result of the disruption of the blood–brain barrier (BBB) [16].

In this case, MRS showed a decrease in the NAA/cr ratio and a slight elevation of cho/cr and lactate peaks in the lesion (Figure 3), suggesting ischemic, demyelinating, or tumor pathology. Lactate is a product of anaerobic glycolysis in the early stage of ischemic stroke and inflammation [17]. Acute demyelinating lesions such as acute disseminated encephalomyelitis (ADEM) or multiple sclerosis (MS) show an increase of choline and lactate, or a reversible decrease of NAA, which suggests axonal damage [18]. However, the persistent state of elevated lactate for 4 months in this case suggests that these lesions were less likely to be caused by ischemia.

Uchiyama et al. [19], Deus-Silva et al. [20], and Skolarus et al. [21] reported similar cerebral lesions after coiling, in which expansive brain swelling was observed after a neurointerventional procedure with hypertonic CM and late-onset allergy-like reaction (LAR) mechanisms [22]. In addition, in all five cases (ours, [19], [20], two cases of [21]), only the lesions that were associated with perfused CAn were described. A previous report showed that multiple small thromboembolic lesions were presented by DWI after coil embolism surgery [23]. Breakdown of the BBB induced by minor ischemic damage in the coiling procedure might be associated with the pathology of these cases. In addition, Skolarus et al. hypothesize that their cases with abnormal white matter changes after cerebral aneurysm treatment may reflect an exaggerated postoperative extravascular inflammatory reaction to the bioactive coils [21]. The bioactive coils consisted of a polyglycolic acid (PGA) filament that was used in our case. Our FDG-PET, MET-PET and ^1^H-MRS findings in this case may explain how the etiology of white matter lesions is suspected of contributing to some kind of inflammation associated with the remote complication of the coiling procedure.

Middleton et al. demonstrated that the degradation time of PGA to complete resorption into a living body is a period of 6 to 12 months [24]. In our case, a follow-up MRI at 6 and 12 months showed a gradual decrease in the initial hyperintense lesions in the territory supplied by the coiled vessel. The transient pathological condition after a coiling procedure, as in our case, might be related with the degradation time of bioactive substances such as PGA.

In conclusion, we present a case of an unusual complication after a coiling procedure. Although it is difficult to identify this etiology without a pathological examination, it is importance to increase awareness of such a potential complication arising from coiling procedures because interventional procedures have become the first choice of treatment for cerebrovascular diseases in many countries.

### Consent

Written informed consent was obtained from the patient for publication of this case report and accompanying images. A copy of the written consent is available for review by the Editor-in-Chief of this journal.

## Abbreviations

ADEM: Acute disseminated encephalomyelitis
BBB: Blood–brain barrier
Cho: Choline
cr: Creatine
CNS: Central nervous system
CAn: Cerebral aneurysm
CSF: Cerebrospinal fluid
CTA: Computed tomographic angiography
CM: Contrast media
CT: Computed tomography
DWI: Diffusion-weighted image
FDG: 2-deoxy-2-[F-18] fluoro-D-glucose
FLAIR: Fluid-attenuated inversion recovery
^1^H-MRS: Proton magnetic resonance spectroscopy
Gd-MRI: Gadolinium-enhanced magnetic resonance imaging
LAR: Late-onset allergy-like reaction
MET: ^11^C-methionine
MRA: Magnetic resonance angiography
MRI: Magnetic resonance imaging
MS: Multiple sclerosis
PET: Positron emission tomography
